# A Life-Stage Approach to Precision Nutrition: A Narrative Review

**DOI:** 10.7759/cureus.66813

**Published:** 2024-08-13

**Authors:** Yeong Sook Yoon, Hye In Lee, Sang Woo Oh

**Affiliations:** 1 Department of Family Medicine, Inje University Ilsan Paik Hospital, Goyang, KOR; 2 Medical Scientific Affairs, Haleon, Seoul, KOR; 3 Department of Family Medicine, Center for Obesity, Metabolism, and Nutrition, Dongguk University Ilsan Hospital, Goyang, KOR

**Keywords:** optimal health, health promotion, age grouping, precision nutrition, life stage nutrition, multivitamin and mineral supplements, healthy aging, nutrition

## Abstract

The concept of precision nutrition highlights the customization of nutrition to specific needs, emphasizing that a one-size-fits-all approach is not sufficient for either optimal nutrition or optimal health. Precision nutrition encompasses a range of factors, from broad strata of age and sex categories to personal characteristics such as lifestyle to an individual’s unique genotype. This breadth of scope requires us to consider how precision nutrition can be implemented in an inclusive and appropriate way for individuals and groups within real-life populations. In this narrative review, we explore the potential of precision nutrition through a life-stage approach that emphasizes age- and gender-specific nutritional needs as these change across the lifespan. Focusing on adult life stages, we delineated trends in age-related conditions and health needs among Korean adults based on national-level survey data (KNHANES 2019-2021). We also reviewed the intake of nutrients associated with these health needs to better understand how life-stage guided approaches to nutrition and supplementation could support optimal health. Looking beyond preventing deficiency or disease, we discuss how tailored supplementation of essential vitamins, minerals, and certain bioactive substances could promote healthy functioning. Finally, we discuss the complexities and challenges of developing multivitamin/multimineral supplements (MVMS) to support life-stage appropriate nutrition while maximizing adherence. Future prospects include leveraging advancements in intelligent technologies and dietary assessments for tracking nutrient intake and health indicators and using these to optimize MVMS formulations in ways that are sensitive to a person’s needs and priorities/preferences at different life stages. By adopting a life-stage guided approach to nutrition, we can better support health and well-being across the lifespan.

## Introduction and background

From infancy through each stage of life, nutrition plays an important role in maintaining healthy functioning and helping to prevent or delay the onset of disease and disability [1-3]. As nutritional requirements change over the life course and also vary between individuals, nutritional recommendations and interventions should be tailored accordingly: one size does not fit all [3-5]. Genetic, environmental, and other factors contribute to inter-individual variability in nutritional requirements and responses to nutritional interventions [3,4,6]. On the other hand, it is also widely documented that subgroups of similar individuals within a population tend to respond in similar ways to nutritional exposures or interventions. With these insights, precision nutrition was introduced as a concept for guiding nutritional recommendations/interventions [4,7]. Although there is currently no universally accepted definition, precision nutrition represents an inclusive, evidence-based approach to tailoring nutritional recommendations/interventions based on biological, environmental, and other factors to optimize health outcomes, e.g., by preventing/delaying disease onset or mitigating disease severity [4,5,7].

**Figure 1:**
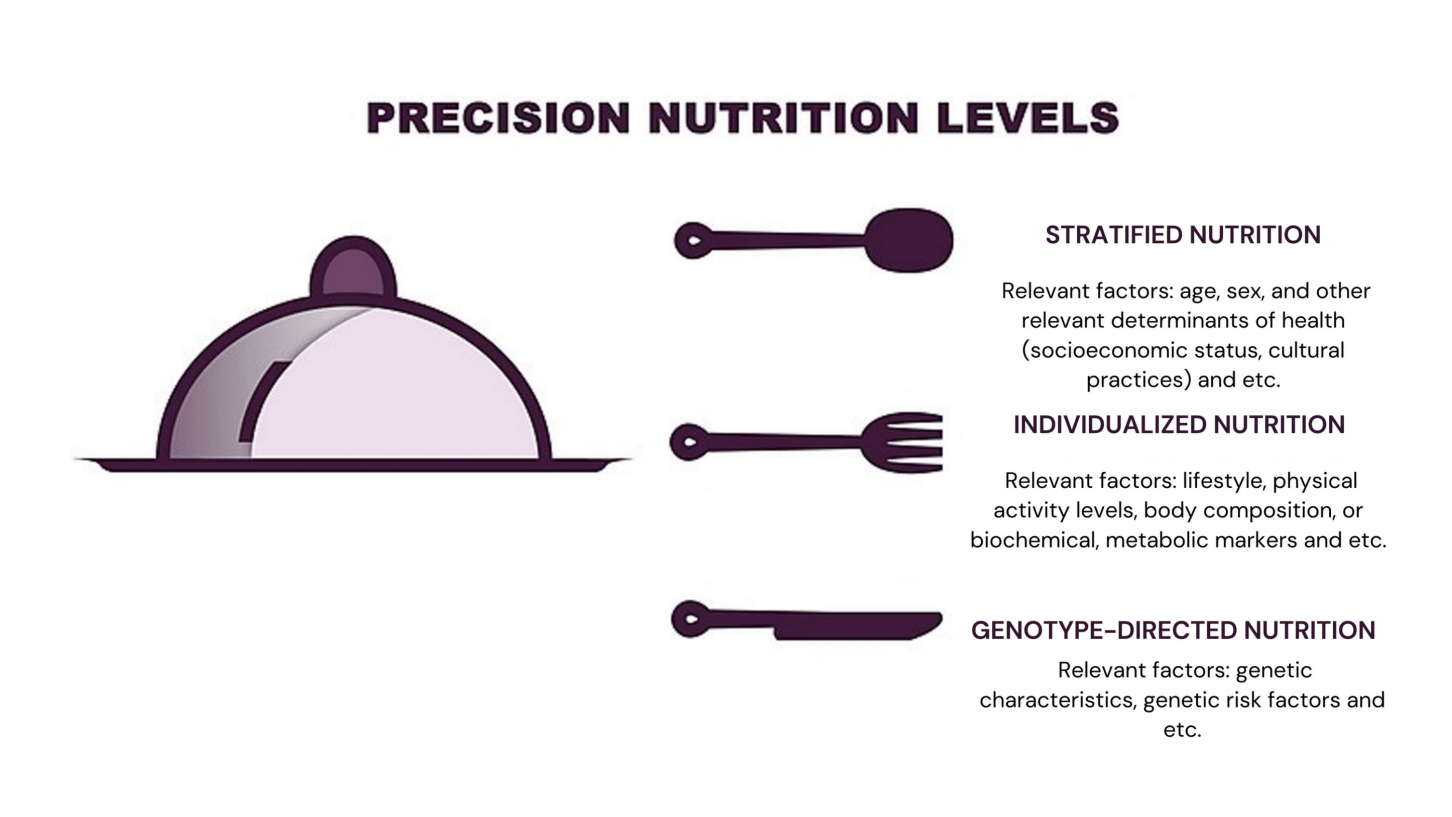
Precision nutrition levels This figure is modified from “Precision Nutrition” by Francesco Pettini, used under CC BY 4.0 via Wikimedia Commons https://creativecommons.org/licenses/by/4.0.

The International Society of Nutrigenetics/Nutrigenomics (ISNN) recognizes three broad levels of precision nutrition (Figure 1): stratified nutrition, individualized nutrition, and genotype-directed nutrition [4]. Stratified nutrition approaches are based upon guidance for population groups defined by factors such as age, sex, and other relevant determinants of health (e.g., socioeconomic status and cultural practices). More precise, individualized nutrition approaches consider additional characteristics such as lifestyle, physical activity levels, body composition, or biochemical and metabolic markers. Genotype-directed nutrition integrates an individual’s genetic characteristics to develop precise, fully personalized recommendations according to their unique patterns of characteristics and risk factors [4].

In this review, we discuss the value of life-stage guided nutrition as a form of precision nutrition that is sensitive to a person’s needs at each life stage. This article explores health needs, age-related conditions, and trends in nutrient intake across life stages, reviews the importance of selected micronutrients in maintaining healthy functioning in each life stage, and discusses future directions for this area. We focus here on life-stage guided precision nutrition to support healthy functioning throughout early, middle, and later adult life.

## Review

Life-stage guided nutrition as a form of precision nutrition

An individual’s requirements for macronutrients and micronutrients to support optimal growth, development, and function will change as they grow, develop, and age [8]. As the importance of life-stage appropriate nutrition in child and adolescent growth and development has been well-described [9,10], this paper focuses on the early, middle, and later stages of adulthood.

Even within defined life stages such as adulthood, nutritional and other health-related needs clearly vary with age. It is, therefore, necessary to consider additional sub-stages that are grounded in biological and other factors. Research by Erikson, Arnett, Lachman and others has delineated additional sub-stages based on biological and psychosocial developmental transitions occurring from early to later adulthood [11-14]. These stages tend to cluster around certain age ranges, albeit with considerable individual variation [11,13,14]. At the population level, age-related patterns of morbidity and health risks can be observed, reflecting changes that affect physical and mental functioning as people age [15]. Therefore, standardized age groupings within identified life stages can facilitate research, planning, and implementation of health initiatives [16,17]. It is acknowledged that stages or sub-groupings might differ from population to population, as well as being influenced by individual variability.

Within Korea, a rapidly aging society where the average life expectancy exceeds 80 years, there is considerable awareness and interest in healthy living. Over the last decade, studies have shown increased awareness and use of dietary supplements and other functional foods as part of individuals’ activities to enhance personal health and well-being [18-20]. Although supplement use has become more prevalent among Korean adults (increasing from 61% who regularly used supplements in 2018 to 75% in 2020), the majority may not be meeting the recommended daily intake levels of several micronutrients, even with dietary supplements [21]. The Korean population thus provides an opportunity to examine possibilities for life-stage guided approaches to diet and supplementation that could help improve nutritional intake and status.

Several studies on health and nutrition in Koreans have utilized sub-groupings corresponding to young, middle, and later adulthood [22-29]. Young adulthood (approximately 19-39 years old) is widely recognized as an important transitional period in which health behaviors and lifestyle patterns strongly influence health and chronic disease risk in later life stages [22,23,27-32]. In middle adulthood (from 40-60 or 65 years old, depending on the definition used), studies indicate an increased prevalence of metabolic syndrome after age 40 and other chronic diseases after age 50 [26,33,34]. To help address a range of health challenges that become prominent in the transitional life-stage beginning at age 40 [35], a national screening program was launched in 2007, targeting adults in this age group [36]. In 2021, the Korean Ministry of Food and Drug Safety published dietary management guidance for “new middle-aged” individuals (50-64 years old) that emphasizes preventive interventions such as healthy eating habits and adequate nutritional intake to prepare for a healthier old age [37]. Considering the above, we note that the 2020 Korean Dietary Reference Intake (KDRI) framework utilizes the following categories for adults: 19-29 years, 30-49 years, 50-64 years, and older adults (65-74 and ≥75 years) [24,25], further supporting the relevance of these age groupings for nutritional policy and interventions in the Korean population.

Dietary guidelines have identified some “nutrients of public health concern” and their associated indicators [38]. While emphasizing healthy dietary patterns, it is also recognized that vitamin/mineral supplements can be useful for individuals who face difficulties in meeting their needs for key micronutrients through food alone [38]. An expert consensus concluded that multivitamin/multimineral supplements (MVMS) can broadly improve micronutrient intakes in individuals/populations where consumption or bioavailability of specific nutrients is insufficient, provided that these supplements contain at least the relevant nutrients in question [39]. Beyond basic supplementation, experts noted that MVMS formulations may also be individualized “according to age, sex, life cycle, and/or other selected characteristics” [39]. Whereas basic MVMS can help individuals improve micronutrient intake to meet their present nutritional needs, more specific life-stage guided approaches to supplementation may be desirable to optimize nutritional status and maintain physiological reserves for life-long health and functioning.

Patterns of health needs and age-related conditions across life-stages in Korean adults

Age is a prominent risk factor for many non-communicable and chronic diseases, the incidence of which increases with age [40,41]. The typical age of onset varies between disease types, potentially resulting from different rates of aging across the body’s organs and systems [42]. Additionally, distinct aging trajectories in men and women have been described, attributed to the effects of hormones and other factors [43,44]. Patterns of disease burden can, therefore, be studied as proxies for health needs and risk factors in men and women across life stages.

To better understand nutrition-related health needs and behaviors across life stages in Korean adults, we undertook a descriptive analysis using nationally representative population health surveillance data for Korea. The Korea National Health and Nutrition Examination Surveys (KNHANES) are conducted periodically to assess health status and health-related behaviors, including food and nutrient intake, in the population. We analyzed KNHANES 2019-2021 data (N=15,556) to delineate trends in common health conditions/health needs and associated nutrient intake among Korean men and women aged ≥19 years.

**Figure 2:**
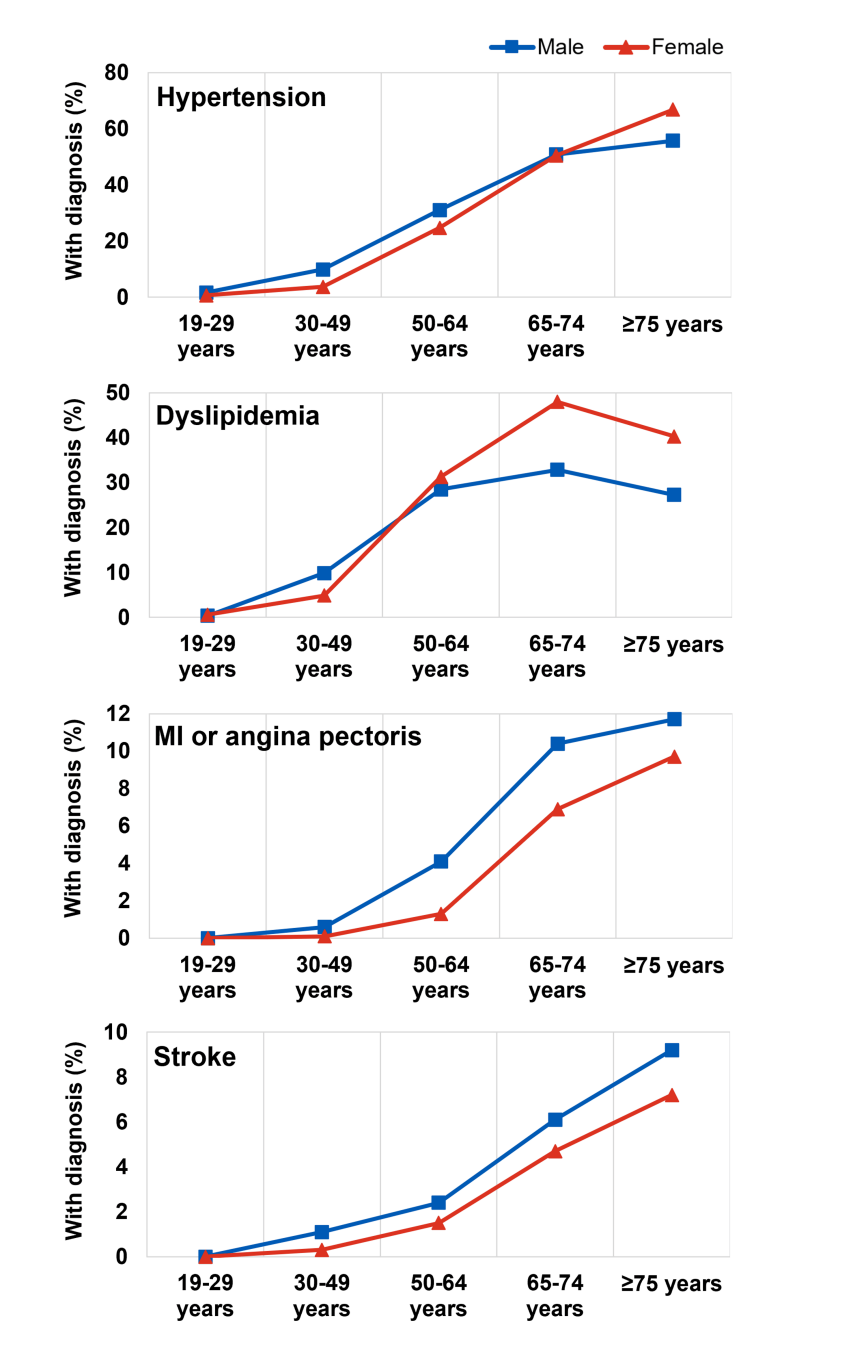
Prevalence of cardiovascular conditions MI, Myocardial Infarction The dataset used for this analysis is obtained from the Korea Disease Control and Prevention Agency (KDCA), https://knhanes.kdca.go.kr/knhanes/eng/index.do.

**Figure 3:**
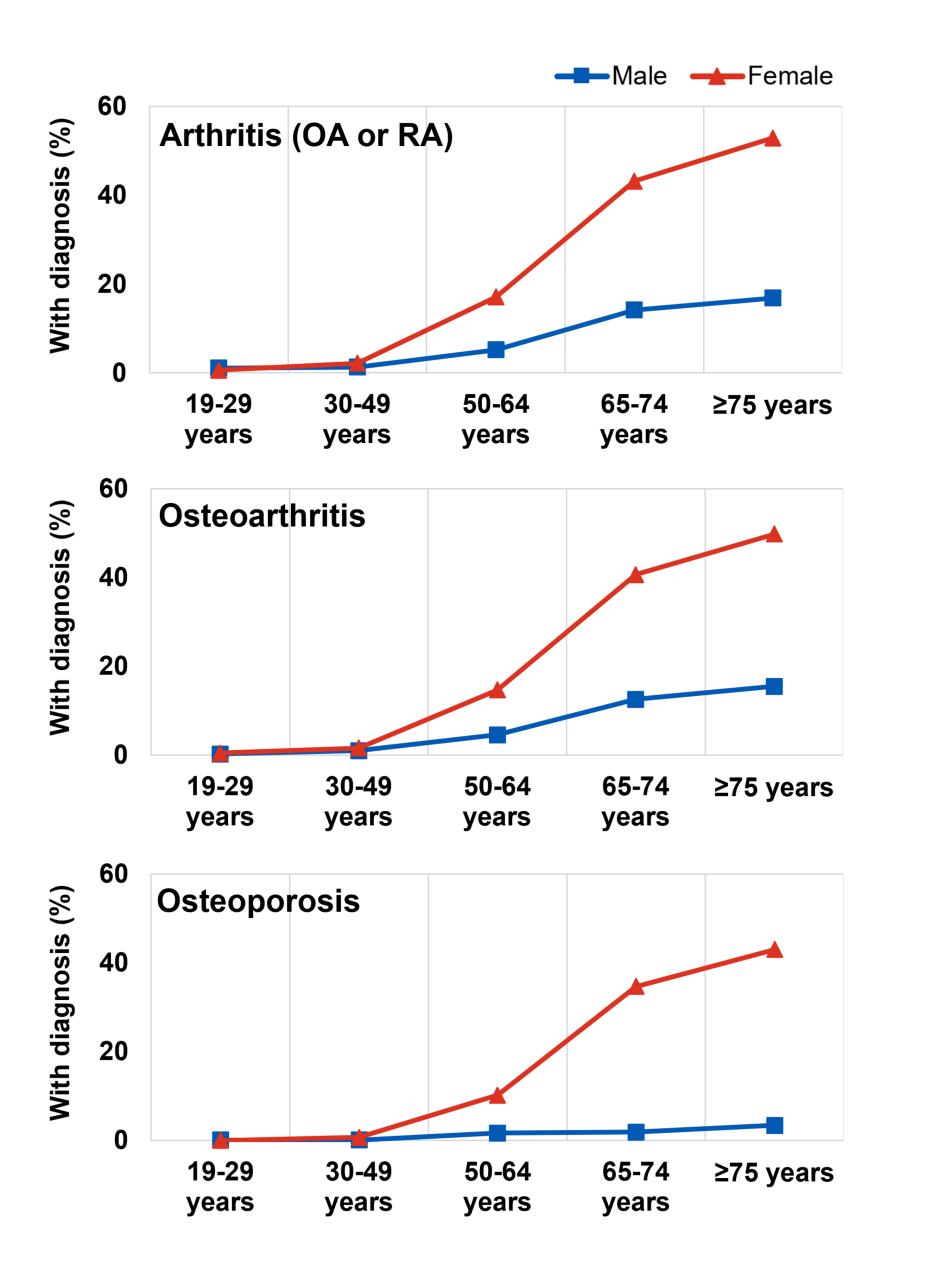
Prevalence of musculoskeletal conditions OA, Osteoarthritis; RA, Rheumatoid arthritis The dataset used for this analysis is obtained from the Korea Disease Control and Prevention Agency (KDCA), https://knhanes.kdca.go.kr/knhanes/eng/index.do.

**Figure 4:**
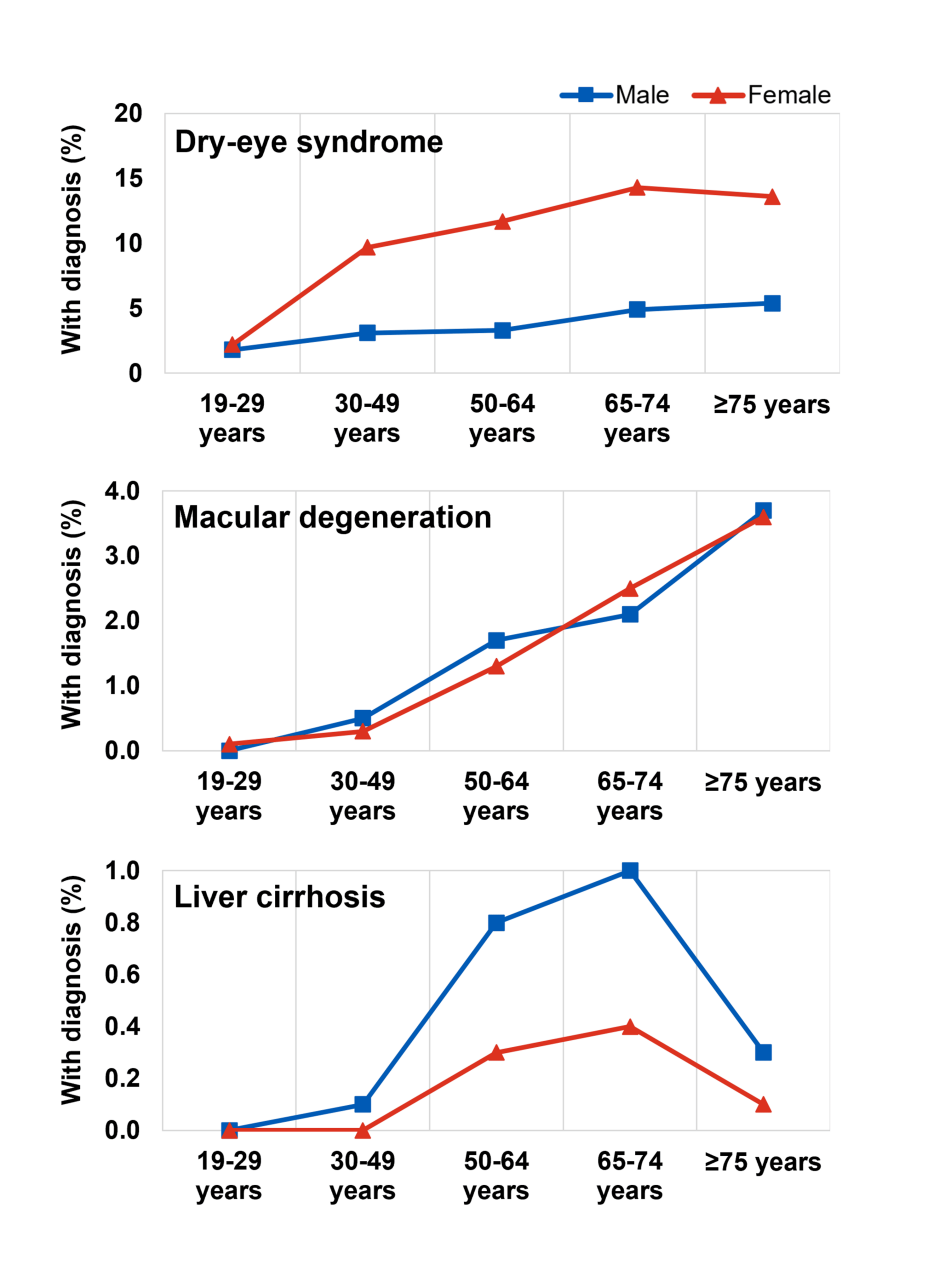
Prevalence of other conditions The dataset used for this analysis is obtained from the Korea Disease Control and Prevention Agency (KDCA), https://knhanes.kdca.go.kr/knhanes/eng/index.do.

We noted clear age-dependent patterns, with various health conditions increasing in prevalence from younger to older age groups (Figures 2, 3, 4). The temporal patterns for some conditions (e.g., dry-eye syndrome, osteoporosis, liver cirrhosis) also differed between men and women. Among the health conditions examined, we noted earlier-onset and later-onset patterns. For example, the prevalence of dry-eye syndrome appeared to increase markedly in women after age 30 (from 2.2% in those 19-29 years old to 9.7% in those 30-49 years old), whereas age-related macular degeneration (AMD) became more common in later life, after age 65. Among cardiometabolic conditions, hypertension and dyslipidemia became more common in men and women from around age 50, and stroke, myocardial infarction (MI) or angina pectoris became more common later in those above 65 years (Figure 2). Musculoskeletal conditions (arthritis, osteoporosis) became more common around the age of 50, especially in women (Figure 3). The prevalence of osteoarthritis was 3.5 times more common in women than in men, with notable differences from around the age of 50.

The observed patterns for these population-level indicators suggest specific and distinct health needs in Korean men and women at various life stages and across major areas of health, e.g., cardiometabolic and musculoskeletal health. Younger men and women (19-29 years old) in Korea typically have a low risk of most common health conditions, except for dry-eye syndrome (especially in women), although the prevalence of dyslipidemia and hypertension starts to increase from age 30 onwards. Both men and women should pay greater attention to bone health from the age of 30 to 40 when bone mass begins to decline [45]. The rate of bone loss increases during mid-life, and after the age of 50, diagnosis of osteoporosis is markedly increased, especially in women around the menopause transition [45]. Recent analyses have highlighted potential calcium and vitamin D deficiency in Korean women aged 30 or older, especially in women over 50 [24,46-48]. Inadequate daily intake of calcium and vitamin D has also been widely reported [49-51]. Therefore, throughout their 40-50s (the peri-menopausal period and the menopause transition), women would benefit from ensuring adequate intake of nutrients that support overall, musculoskeletal and cardiometabolic health. Together with maintaining a healthy body weight and levels of physical activity, this could help reduce the impact of menopausal symptoms [52,53].

For men in Korea, supporting cardiometabolic health is likewise important, with dyslipidemia and hypertension prevalence starting to increase gradually from as early as age 30, and stroke and MI/angina pectoris prevalence increasing markedly above the age of 50 (Figure 2). Cardiovascular conditions were also more common among men than women in each age group. The notable increase in liver cirrhosis prevalence in men above age 30 (Figure 4) highlights the need for attention to liver health among young adults since the effects of liver damage can take ≥10 years to manifest [54,55].

Trends in nutrient intake among Korean adults

**Figure 5:**
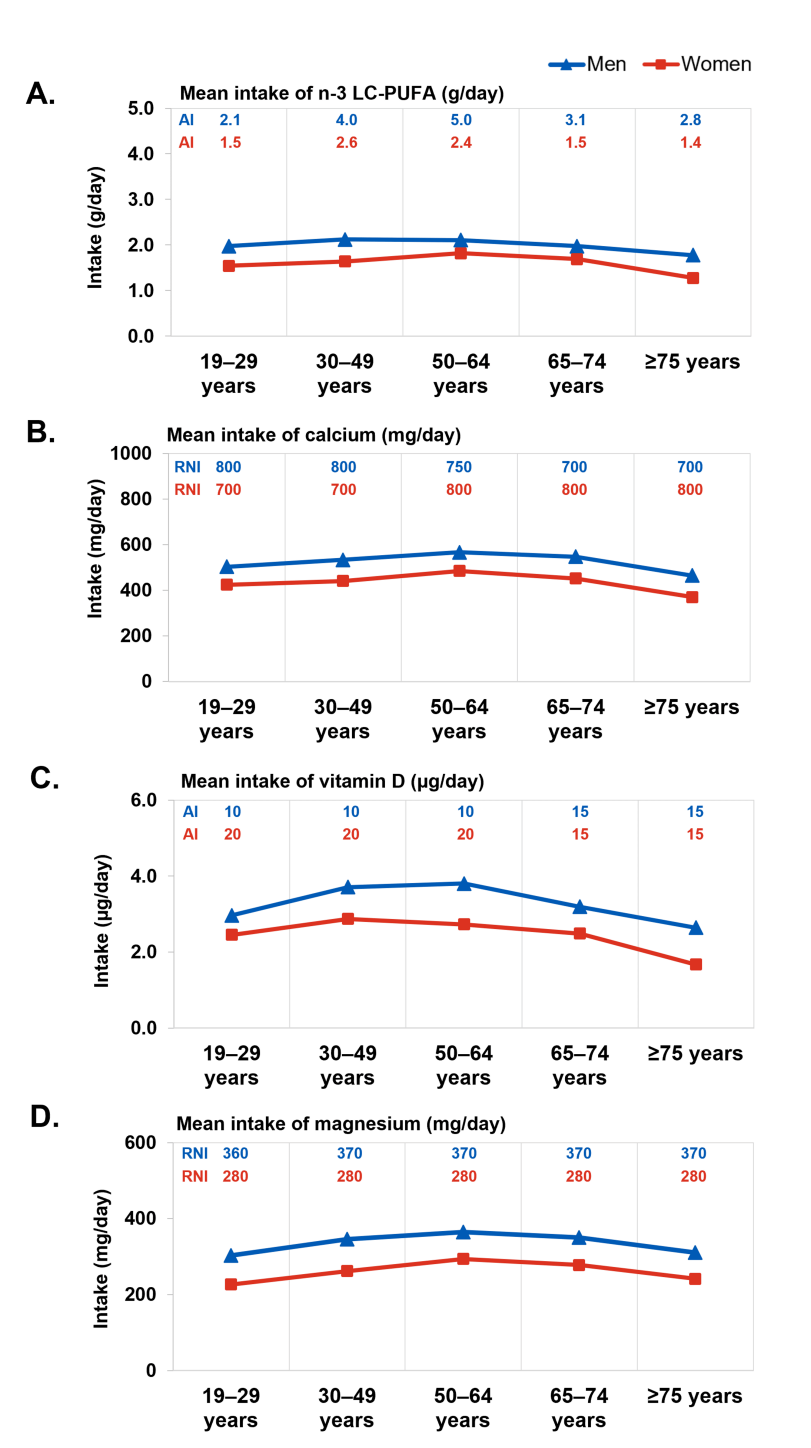
Reported intake of important nutrients n-3 LC PUFAs, n-3 Long-chain polyunsaturated fatty acids; AI, Adequate intake; RNI, Recommended nutrient intake. Red lines represent women and blue lines represent men. A. Mean daily intake of n-3 LC-PUFAs (g/day) among men and women across different age groups. The adequate intake (AI) levels are indicated for each age group; B. Mean daily calcium intake in mg/day among men and women across different age groups. The recommended nutrient intake (RNI) is indicated for each age group; C. Mean daily intake of vitamin D in µg/day among men and women across different age groups. The adequate intake (AI) levels are indicated for each age group; D. Mean daily intake of magnesium in mg/day among men and women across different age groups. The recommended nutrient intake (RNI) levels are shown for each age group. The dataset used for this analysis is obtained from the Korea Disease Control and Prevention Agency (KDCA), https://knhanes.kdca.go.kr/knhanes/eng/index.do.

In view of the potential age group-specific health needs suggested by our analysis, we examined the intakes of nutrients known to be important for these areas of health in the KNHANES data over the same period (2019-2021). Based on mean daily intakes of nutrients in comparison with the corresponding estimated average requirement (EAR), recommended nutrient intake (RNI) or adequate intake (AI) levels, a substantial proportion of men and women may have inadequate intake of micronutrients, including calcium, magnesium, omega-3 long-chain polyunsaturated fatty acids (n-3 LC-PUFAs), and vitamin D. Intake levels below EAR (calcium and magnesium) and AI levels (n-3 LC-PUFAs and vitamin D) may be considered inadequate. Among both men and women, daily intake of n-3 LC-PUFAs, calcium, vitamin D, and magnesium remained relatively constant across life stages or slightly decreased in older age groups (Figures 5A-5D). The requirements for a number of nutrients remain relatively constant or even increase with age (Table 1) [25], suggesting opportunities for emphasizing life-stage approaches to improve nutritional intake in the population.

**Table 1:** 2020 dietary reference intakes for Koreans (Ministry of Health and Welfare, Korea) EAR, estimated average requirement; RNI, recommended nutrient intake; AI, adequate intake; UL, upper intake level; EPA, eicosapentaenoic acid; DHA, docosahexaenoic acid. These reference values include estimated average requirement (EAR), recommended nutrient intake (RNI), adequate intake (AI), and tolerable upper intake level (UL). From: 2020 Dietary Reference Intakes for Koreans [25]. a. Only for non-food magnesium sources.

	Age group	Calcium (mg/day)				Vitamin D (µg/day)				Methionine (g/day)				Magnesium (mg/day)				EPA+DHA (mg/day)			
		EAR	RNI	AI	UL	AI	UL	AI	UL	EAR	RNI	AI	UL	EAR	RNI	AI	UL^a^	EAR	RNI	AI	UL
Males	19-29	650	800	-	2,500	-	-	10	100	1.0	1.4	-	-	300	360	-	350	-	-	210	-
	30-49	650	800	-	2,500	-	-	10	100	1.1	1.3	-	-	310	370	-	350	-	-	400	-
	50-64	600	750	-	2,000	-	-	10	100	1.1	1.3	-	-	310	370	-	350	-	-	500	-
	65-74	600	700	-	2,000	-	-	15	100	1.0	1.3	-	-	310	370	-	350	-	-	310	-
	≥75	600	700	-	2,000	-	-	15	100	0.9	1.1	-	-	310	370	-	350	-	-	280	-
Females	19-29	550	700	-	2,500	-	-	10	100	0.8	1.0	-	-	230	280	-	350	-	-	150	-
	30-49	550	700	-	2,500	-	-	10	100	0.8	1.0	-	-	240	280	-	350	-	-	260	-
	50-64	600	800	-	2,000	-	-	10	100	0.8	1.1	-	-	240	280	-	350	-	-	240	-
	65-74	600	800	-	2,000	-	-	15	100	0.7	0.9	-	-	240	280	-	350	-	-	150	-
	≥75	600	800	-	2,000	-	-	15	100	0.7	0.9	-	-	240	280	-	350	-	-	140	-

In men and women 50-64 years and older, average calcium intake was lower even as RNI increased (Figure 5B); the gap between average intake and requirements was larger than for younger age groups, especially for women in post-menopausal life stages. Overall, these observations are consistent with the “nutritional problem” of inadequate calcium intake highlighted in recent analyses that informed the 2020 KDRI [24]. Magnesium intake levels were generally above EAR but below RNI across most age groups (Figure 5D). In women ≥50 years, average magnesium intakes further decreased slightly although RNI remains the same as for younger women (280 mg/day). In men, n-3 LC-PUFA intake was generally below AI across age groups, especially among those ≥50 years (Figure 5A). Average n-3 LC-PUFA intake also declined slightly among women ≥65 years, suggesting a need for life-stage guided nutrition to help maintain adequate intake as people age. Vitamin D intake was considerably below AI across various age groups, especially for older adults (Figure 5C). Given these indications that inadequate micronutrient intake is common among Korean adults, dietary interventions, including supplementation, may be warranted to improve overall nutrient intake and reduce the prevalence of nutrient inadequacy. In the following sections, we discuss the significance of these nutrients in the areas of health needs identified.

Nutrients for cardiovascular health 

n-3 LC-PUFAs

As n-3 LC-PUFAs are integral components of cell membranes and other important biological structures, they are involved in many critical physiological processes [56]. Mechanistic and clinical data support cardiovascular (CV) protective effects of n-3 LC-PUFAs, especially docosahexaenoic acid (DHA) and eicosapentaenoic acid (EPA), through a number of pathways [56-59]. DHA and EPA have been found to favorably influence levels of serum lipid parameters as well as tissue inflammatory markers [56-58,60]. Down-regulation of inflammation-related genes and attenuation of pro-inflammatory signaling by EPA/DHA are thought to contribute to these CV-protective effects [58,61]. Favorable effects of n-3 LC-PUFAs on blood pressure (BP) and endothelial function have also been described [62,63].

Although randomized controlled trials (RCTs) have not consistently shown effects on major adverse CV events (MACE) such as MI or coronary heart disease (CHD), it has been noted that differences in several factors (type/dose of n3 LC-PUFAs studied, the comparator interventions used, as well as the populations studied) may partly explain these inconsistencies [58,59,64]. It should also be noted that many of the studies were conducted in populations with CVD and/or risk factors (often middle-aged or older adults), making it challenging to determine the implications of findings for supplementation in healthy individuals. A 2021 meta-analysis/meta-regression study conducted using 40 RCTs of EPA/DHA supplementation for CVD prevention (n=135,267) concluded that supplementation was associated with reduced risk of MI (risk ratio (RR): 0.87, 95% confidence interval (CI) 0.80-0.96), CHD events (RR, 0.90, 95% CI 0.84-0.97), and CHD mortality (RR: 0.91, 95% CI 0.85-0.98) [65]. This analysis also suggested that protective effects might increase with supplement dosage. A 2023 systematic review including 17 major RCTs for MACE prevention and residual CV risk reduction (n=143,410) concluded that n-3 LC-PUFA supplementation had significant beneficial effects on CV death (RR 0.94; 95% CI: 0.88-0.99) and fatal/non-fatal MI (RR: 0.83; 95% CI: 0.72-0.95), with generally good safety and tolerability [58]. This analysis also suggested that EPA may have stronger CV-protective effects than DHA; the authors comment that, although DHA demonstrated limited CV benefit, its effects could be more closely linked to neuroprotection [58].

Based on the favorable effects of n-3 LC-PUFAs on CV risk factors, vascular function, and inflammatory markers, and the lack of adverse effects when consumed at recommended levels, guidelines for the general population recommend intake of n-3 LC-PUFAs of at least 250 mg/day. In most cases, this is defined in terms of adequate intake (AI) levels, due to insufficient evidence for defining EARs or RNIs for healthy individuals. Recommended adequate intake of n-3 LC-PUFAs varies depending on the country/region and for specific population groups. Internationally and in the US and Europe, daily intakes of at least 250 mg/day n-3 LC-PUFA or ≥2 servings/week of oily fish are recommended across adult age groups, emphasizing the importance of this nutrient at all stages of life [38,66,67]. In the 2020 KDRIs, AI levels range from 150-240 mg/day for women and 210-500 mg/day for men, depending on age group [25,68]. It should be noted that, although these intake levels were proposed based on cardiovascular considerations for healthy adults, there is emerging evidence that higher levels of n-3 LC-PUFAs may be beneficial for supporting healthy cognitive function (discussed below in the section on nutrients for cognitive function). Further research is required to clarify variations in n-3 LC-PUFA requirements for different aspects of health across adult age groups and to inform updates to dietary recommendations as needed [68,69].

Nutrients for bone, muscle and joint health 

Calcium and Vitamin D

Together, calcium and vitamin D play crucial roles in bone health; individually, both also have other essential physiological functions throughout the body, including muscle function and immune system support [70,71]. Besides being a core mineral constituent of bones, calcium is also essential for muscle contraction, synaptic transmission, and hormone secretion [71]. Calcium homeostasis is therefore critical and depends on vitamin D, which regulates dietary calcium absorption, renal calcium reabsorption, and calcium release through bone remodeling [72]. Optimal calcium metabolism thus requires adequate and balanced intake of both calcium and vitamin D. Abnormal bone remodeling due to several factors (including age, low sex hormone levels, low calcium/vitamin D intake, etc.) could result in metabolic bone diseases such as osteoporosis [73].

Calcium must be obtained from dietary sources such as dairy, calcium-fortified foods, or supplement products. Although vitamin D can be synthesized when the skin is exposed to ultraviolet B radiation from sunlight, diet is an important source of vitamin D, especially for individuals with insufficient sun exposure. Dietary requirements for calcium and vitamin D may vary among individuals according to the need for bone development and maintenance across life stages. The recommended daily allowance (RDA) represents the amount considered necessary to meet the nutritional needs of most (97-98%) healthy individuals. Table 2 shows current US RDAs for calcium intake across different age groups. The RDA of 1000 mg for adults aged 19-50 is intended to support bone maintenance and achieve a neutral calcium balance. The RDA increases to 1200 mg for males >70 years and for females >50 years [74]. This reflects women’s increased calcium requirements after menopause. Estrogen promotes efficient calcium absorption and renal conservation, and both deteriorate in the estrogen-deprived post-menopausal state. Declining levels of estrogen and other sex hormones also increase bone remodeling and loss due to an imbalance between osteoclast and osteoblast activity. Hence, calcium intake should be maintained or increased in post-menopausal women and older men to mitigate the potential impact on bone health.

**Table 2:** Recommended dietary allowances (RDAs) for calcium in US adults From: Dietary Reference Intakes for Calcium and Vitamin D [74].

Age	Male	Female
19–50 years	1,000 mg	1,000 mg
51–70 years	1,000 mg	1,200 mg
>70+ years	1,200 mg	1,200 mg

In general, RDAs for vitamin D are aimed at supporting bone health and maintaining normal calcium metabolism in healthy individuals (Table 3) [74]. RDAs are conservatively based on the assumption of minimal sun exposure, as sunlight may not be a major source of vitamin D for many individuals in high-latitude regions [38]. Guidelines for vitamin D intake and supplementation vary across countries and settings (e.g., professional society guidelines), likely reflecting the purpose of the guidelines and/or methodologies used to establish them. For the general population in the United Kingdom (individuals aged ≥4 years), intakes of 10 µg/day (400 international units (IU)/day) are considered sufficient [75], whereas US RDAs recommend 15 µg/day in adults 19-70 years old (Table 3). On the other hand, the Endocrine Society recommends daily supplemental vitamin D doses of 37.5-50 µg (1,500-2,000 IU) for adults to maintain serum 25(OH)D levels above 75 nmol/L (30 ng/mL) [76]. Although recommended vitamin D intake levels remain relatively constant across adult age groups (slightly increased for older adults; Table 1 and Table 3), calcium metabolism and requirements may change across life stages, especially in peri-menopausal and post-menopausal women. It is thus important to pay attention to vitamin D intake and status to support a healthy calcium balance.

**Table 3:** Recommended dietary allowances (RDAs) for vitamin D in US adults IU, International units. From: Dietary Reference Intakes for Calcium and Vitamin D [74].

Age	Male	Female
19-50 years	15 µg (600 IU)	15 µg (600 IU)
51-70 years	15 µg (600 IU)	15 µg (600 IU)
>70 years	20 µg (800 IU)	20 µg (800 IU)

Vitamin D and calcium intake recommendations for Korean adults are summarized in Table 1 [25]. Among Korean women, calcium intake is reportedly generally well below the DRI, and vitamin D insufficiency is also very common in the general population [77]. These are consistent with trends noted in our analysis of KNHANES data (Figures 5B, 5C). Additionally, the average age of menopause in Korea is 49.4 years, and the proportion of Korean women experiencing early or premature menopause appears slightly higher than in other regions. A cross-sectional study analyzing data from the US and Korea reported that 2.8% of Korean women experience premature menopause and 7.2% experience early menopause. In comparison, 1.7% of US women experience premature menopause and 3.4% experience early menopause [78]. These trends emphasize the importance of adequate calcium intake for women, starting from their 40s, to support bone health through menopause and beyond.

Studies on the effects of calcium and vitamin D supplementation have yielded mixed conclusions. However, it should be noted that such studies vary considerably in terms of the populations and outcomes examined, whether or not calcium and vitamin D were supplemented together, as well as the duration, doses and forms of supplementation. This implies it is important to tailor calcium/vitamin D supplementation to the needs of individuals based on factors such as their life stage, health status, and lifestyle patterns.

A systematic review and meta-analysis in young adults (20-35 years old) found that calcium supplementation significantly improved bone mineral density (BMD) and bone mineral content, particularly at the femoral neck [79]. Additionally, supplementation around or before the time of peak bone mass had a more pronounced effect. In considering bone health across the lifespan, the authors concluded that the intervention window for calcium supplementation should start around/before the age when bone mass peaks (20-35 years). A systematic review of seven studies in pre-menopausal women (18-42 years old; n=941) found no significant differences in BMD (intervention versus placebo) for groups receiving calcium, vitamin D, or combined calcium/vitamin D supplementation [80]. A 2015 meta-analysis of eight RCTs in older adults (n=30,970) found that calcium plus vitamin D supplementation resulted in 15% risk reduction for total fractures (summary relative risk estimate (SRRE): 0.85, 95% CI, 0.73-0.98) and 30% risk reduction for hip fractures (SRRE: 0.70, 95% CI 0.56-0.87) [81]. Another systematic review of nine studies in men and women ≥65 years (n=49,853) reported that taking vitamin D alone, in the forms tested in the studies, did not reduce bone fracture risk. However, with combined vitamin D/calcium supplementation, there were slight reductions in hip and other fractures, with a relative risk of 0.84 (95% CI 0.74-0.96) [82]. Similarly, a meta-analysis of 33 studies (n=83,083) found no significant effect of oral vitamin D supplementation alone on total fracture risk. However, vitamin D3 (700-800 IU/day) plus calcium significantly reduced total, hip, and non-vertebral fractures, particularly in females, highlighting the importance of balanced vitamin D and calcium supplementation [83].

A 2022 meta-analysis of seven studies in older adults reported that daily oral supplementation (800 IU vitamin D3, 1200 mg calcium) reduced hip and non-vertebral fractures, albeit without significantly increasing femoral neck BMD [84]. The study suggested several implications for practice, highlighting that vitamin D and calcium are not the only elements required for bone health and fracture prevention, and advocating a holistic approach to supplementation. This may include efforts to promote and maintain musculoskeletal health from an early age, with proper nutrition to optimize calcium and vitamin D3 intake, and a healthy lifestyle with adequate sunlight exposure and regular exercise [84].

Methylsulfonylmethane (MSM)

Sulfur is an essential component of important biomolecules, including amino acids, hormones, vitamins, and coenzymes [85]. Dietary protein, specifically the sulfur-containing amino acids cysteine and methionine, is the main source of sulfur [85] Although RDAs are unavailable, guidelines recommend methionine and cysteine intakes of 14 mg/kg body weight. These recommendations are based primarily on nitrogen balance studies, potentially underestimating actual requirements for sulfur in the body [86,87]. Considering the biological importance of this nutrient, sulfur-containing dietary supplements may be relevant if dietary intake is inadequate (Table 1), for example, among individuals with a strict vegetarian diet. One such dietary supplement is MSM, a naturally occurring sulfur-containing compound widely taken for maintenance of joint and cartilage health, and sometimes prescribed to osteoarthritis patients. MSM is thought to exert its effects through its anti-inflammatory, antioxidant, and immunomodulatory activities [88].

Available studies suggest some favorable effects of oral MSM supplementation on outcomes related to joint health and function, although more research is required [89]. A pilot trial randomized 50 adults with knee OA, aged 40-76 years, to receive twice-daily oral MSM (total 6.0 g/day) or placebo for 12 weeks [90]. Improvements in the Western Ontario and McMaster Universities Osteoarthritis Index (WOMAC) at Week 12 were significantly larger with MSM versus placebo (p=0.041 and p=0.045 respectively, for pain and physical function) [90]. MSM also improved activities of daily living on the SF-36 evaluation [90]. An RCT in 49 adults (45-90 years with radiographically confirmed knee OA) investigated the effects of a lower total daily dose (3.375 g/day) of MSM versus placebo for 12 weeks [91]. MSM supplementation significantly improved WOMAC physical function and total symptom scores (p=0.04 and p=0.03, respectively) versus placebo [91]. A 2023 RCT in healthy adults (aged 26-85; n=8) with mild knee pain found significantly greater improvement in Japanese Knee Osteoarthritis Measure total scores with MSM versus placebo (p=0.046) at 12 weeks [92]. MSM consumption was also associated with improved overall health condition ratings (p=0.032). Taken together, these findings imply the potential of MSM as a supplement for joint health and function, especially for women, who show increasing prevalence of OA and arthritis in middle age or earlier (Figure 3). This must be substantiated through additional research.

Magnesium

Magnesium regulates numerous physiological processes in the body, from energy metabolism to muscle and nervous system function [93]. Together with calcium, magnesium plays a central role in the muscle contraction cycle, being required for calcium re-uptake in the sarcoplasmic reticulum [94]. Insufficient levels of extracellular magnesium can result in hypercontractility, manifesting as muscle cramps and spasms [95]. As magnesium also supports proper synaptic transmission at the neuromuscular junction, low extracellular magnesium can increase excitatory postsynaptic potential and neuromuscular hyperexcitability [95].

Magnesium is essential throughout life stages, and recommended magnesium intake varies depending on gender and age group. Table 1 shows recommended magnesium intake levels for Korean adults [25]. The EAR for magnesium is 10 mg higher for women >31 years (240 mg) compared with younger women (19-30 years). Current US RDAs similarly indicate that magnesium requirements change with age, increasing after age 30 (Table 4; [96]) The current RDAs for US adults similarly indicate that magnesium requirements change with age, increasing after age 30 (Table 4). RDAs range from 310-320 mg/day for women and 400-420 mg/day for men.

**Table 4:** Recommended dietary allowances (RDAs) for magnesium in US adults From: Dietary Reference Intakes: Calcium, Phosphorus, Magnesium, Vitamin D and Fluoride [96].

Age	Male	Female
19–30 years	400 mg	310 mg
31–50 years	420 mg	320 mg
51+ years	420 mg	320 mg

Studies on magnesium supplementation for muscle health have examined the effects on muscle pain and function in clinical and healthy populations. A 2017 meta-analysis assessed the effects of magnesium supplementation on muscle fitness across 14 RCTs targeting three different populations: athletes or physically active individuals (n=215; mean age: 24.9 years), untrained healthy individuals (n=95; mean age: 40.2 years), and elderly or alcoholic individuals (n=232; mean age: 62.7 years). Magnesium supplementation had the most pronounced beneficial effects in elderly individuals, leading to the conclusion that supplementation may be most beneficial for individuals at risk of or with magnesium deficiency, such as the elderly [97]. A study on pre-menopausal women with fibromyalgia (a condition that causes widespread muscle pain, with possible links to deficiencies in minerals like magnesium and calcium [98]) reported that magnesium citrate supplementation was associated with significant improvement in fibromyalgia symptom indices and impact (muscle tenderness, fibromyalgia impact questionnaire, Beck depression scores) [99]. An RCT involving 139 healthy older women (mean age: 71.5 years) showed significantly improved physical performance (Short Physical Performance Battery, sit-stand, and walking speed tests) after 12 weeks of magnesium supplementation (300 mg/day) versus no intervention (n=77). This supports the potential benefits of magnesium supplementation in maintaining physical functioning.

Lastly, magnesium has also been found to improve inflammation. This is of clinical relevance, as the phenomenon of chronic low-grade inflammation observed in older individuals (often termed "inflammaging") is strongly implicated as a risk factor for frailty, morbidity, and mortality. A meta-analysis of 17 RCTs (n=889) found that magnesium supplementation significantly improved serum inflammatory markers (decreased C-reactive protein and increased nitric oxide levels), suggesting potential benefits in mitigating the effects of inflammaging [100].

Nutrients for eye health

Lutein

Three isomeric carotenoid pigments found in the macula, lutein, zeaxanthin and meso-zeaxanthin, are essential for optimal retinal function, especially color vision [101]. The antioxidant properties of lutein and zeaxanthin help neutralize reactive oxygen species generated by light or other oxidative stressors, helping to protect the retina from photooxidative damage [102]. In normal eyes, higher macular pigment levels have been associated with better visual performance and enhanced visual acuity [103].

Studies suggest that macular lutein and zeaxanthin levels may decline significantly with advancing age [104]. A cross-sectional study in Korean adults aged 30-79 years reported that macular pigment optical density (MPOD) significantly decreased with age (p=0.008) [105]. The mean estimated MPOD was significantly lower in adults with dry AMD compared with normal healthy adults aged >50 years (p=0.001) [105]. It was reported that high-dose lutein supplementation can help maintain macular pigment density: among older individuals (61-84 years old) with dry AMD, those who regularly consumed high-dose lutein supplements had macular carotenoid levels similar to their healthy peers, whereas those who did not use lutein supplements had significantly lower macular carotenoid levels [104]. The effects of lutein supplementation on macular MPOD in patients with AMD have also been evaluated in RCT settings [106]. The LISA study randomized 126 AMD patients (stages II-IV) aged 50-90 years to receive oral lutein (20 mg/day from Months 1-3, 10 mg/day from Months 4-6) or placebo for 6 months [106]. Lutein supplementation for 6 months resulted in significantly higher increases in MPOD versus placebo (percent difference from baseline: 27.9% vs 2.9%; p<0.001) [106]. Notably, increased MPOD was significantly associated with improvement in both macular function and visual acuity at 6 months (p=0.027 and p=0.013, respectively) [106].

Lutein and other macular pigments are obtained exclusively from carotenoid-rich dietary sources, including fruits, vegetables, and egg yolks [101]. Although existing dietary guidelines do not include intake recommendations for lutein, studies suggest that intakes of approximately 6 mg per day are associated with a reduced risk of AMD [107,108]. Additionally, the Age-Related Eye Disease Study 2 RCT found that supplementation with lutein (10 mg/day) and zeaxanthin (2 mg/day) could reduce progression to advanced AMD in individuals with low dietary intake of these nutrients [109]. These clinical findings suggest that lutein intake may be helpful for individuals throughout their 40s and 50s to maintain retinal health.

The focus of DRIs has traditionally been on preventing nutrient deficiency and toxicity. However, there is growing interest in extending such forms of guidance to include non-essential bioactive substances that could promote overall health and reduce the risk of specific diseases. Lupton et al. outlined nine criteria to determine the suitability of a bioactive substance for DRI-like recommendations [110]. Lutein meets these criteria, suggesting that it is ready for consideration in intake recommendations. Establishing dietary guidance for lutein could encourage the inclusion of lutein-rich foods in daily diets and increase awareness of its potential benefits for supporting visual health across various life stages.

n-3 LC-PUFAs for Eye Health

Some studies suggest a potential role for n-3 LC-PUFAs in managing DES, attributed to their anti-inflammatory activity. An RCT in individuals with contact lens-induced DES showed that n-3 LC-PUFA supplementation significantly improved DES symptoms, tear production and tear film stability (TFS), as well as conjunctival morphology, relative to placebo [111]. In another RCT, oral supplementation with n-3 LC-PUFA 1.2 g/day (480 mg DHA, 720 mg EPA) for 3 months significantly improved DES symptoms, tear production and TFS compared with placebo in adults with symptomatic computer vision syndrome (CVS) [112]. A 2019 meta-analysis (17 RCTs) of 3,363 adults with DES of various etiologies found that n-3 LC-PUFA supplementation (fish oil supplements with daily doses from 127.5-2,000 mg for EPA and 99-1,000 mg for DHA) resulted in significantly greater improvement in DES symptoms compared with placebo (p<0.001) [113]. Both tear production and TFS were also significantly improved with n-3 LC-PUFA supplementation versus placebo [113].

Nutrients for cognitive health

n-3 LC-PUFAs for Cognitive Health

n-3 LC-PUFAs are important structural and functional biomolecules, especially in the brain, and supplementation may be relevant to support cognitive as well as cardiovascular health. Indeed, several studies have demonstrated that n-3 LC-PUFA supplementation can influence brain structure and potentially cognitive function. A 2022 systematic review of nine studies reported that high-intensity n-3 LC-PUFA supplementation appears to improve cognitive performance, consistent with previous studies suggesting that DHA intake may help improve early deficits in memory and learning related to cognitive aging [114]. The included studies demonstrated the potential benefits of n-3 LC-PUFA supplementation on cognitive performance in different age groups, including younger and older participants [114]. The review concluded that 900 mg of DHA may be useful as a dietary neuroprotective agent for specific types of early cognitive impairment, and highlighted US recommendations for consuming 3 g of n-3 LC-PUFA daily (up to 2 g/day from dietary supplements). Systematic reviews of limited studies in older adults with mild cognitive impairment (MCI) suggest some benefits in global cognition [115]. A systematic review and dose-response meta-analysis (24 studies, n=9660) on studies of n‑3 LC-PUFA for cognitive function in middle‑aged or older adults without dementia reported potential benefits in cognition (specifically executive function) increasing up to 12 months of supplementation [116]. This trend was more prominent with daily n3 LC-PUFA intakes exceeding 500 mg, and in populations where blood n-3 LC-PUFA levels were not very low. Based on the findings, the authors suggest that n-3 LC-PUFA intake recommendations, which are currently based mainly on CV considerations, could be enhanced by incorporating considerations for cognitive health [116].

Other nutrients and bioactive compounds with the potential to support life-stage nutrition

Traditionally, discussions of nutrition and nutrients have focused on “essential” nutrients, including macronutrients, vitamins and minerals that are needed to maintain health and support normal growth and development [117,118]. Many of these nutrients were identified through their association with “deficiency” diseases. On the other hand, a growing body of evidence supports the potential of bioactive compounds and functional foods, alongside an adequate and healthy diet, for supporting optimal health and well-being. This goes beyond the prevention of deficiency diseases and encompasses concepts such as protective effects in age-related and chronic health conditions. Compounds such as PUFAs (discussed above), hyaluronic acid, plant flavonoids and polyphenols such as those found in milk thistle or saw palmetto extract, and octacosanol, though not conventionally recognized as nutrients, have demonstrated biological activity in supporting and enhancing physiological function. These bioactive substances, such as hyaluronic acid, milk thistle, saw palmetto extract, and octacosanol, have shown promising potential for supporting various aspects of healthy aging, complementing the foundational role of essential nutrients. For example, oral intake of hyaluronic acid (100-200 mg) for 12 weeks improved skin hydration from as early as Week 2 across all skin types in both younger and older adults [119]. Milk thistle contains silymarin, a flavonoid compound reported to have hepatoprotective properties [120]. A 2024 meta-analysis showed that silymarin decreased serum lipids and liver enzyme levels in patients with nonalcoholic fatty liver disease, thereby significantly improving liver histology [121]. Such properties could be beneficial for individuals who may be at greater risk of liver injury, e.g., due to lifestyle factors such as heavy alcohol consumption, which is reportedly common among younger men in Korea. Saw palmetto extract is a traditional herbal remedy used to treat lower urinary tract symptoms (LUTS) associated with benign prostatic hyperplasia (BPH), a common age-related condition described in approximately 50% of men in their 50s, increasing to 90% among men over 80 [122]. In men with BPH-associated LUTS, treatment with saw palmetto extract has been reported to improve symptom scores, although definitive studies with standardized extracts are needed to demonstrate consistent efficacy [123,124].

Future directions and opportunities for life-stage nutrition

In this review, we have explored a life-stage guided approach to precision nutrition, supported by supplementation of important vitamins, minerals and other bioactive substances with the potential to support optimal health beyond preventing disease. To adopt life-stage guided nutrition approaches, we need to consider changing needs due to growth and development, as well as age-related changes in metabolism, nutrient absorption, and tissue/organ function through early, middle, and later adult life. As earlier events contribute to individuals’ health and aging trajectories, including the risk of developing age-related conditions [125], understanding how these can be modified by nutrition is essential for targeted and effective interventions at each life stage. Tailoring supplementation strategies from the starting point of age and gender strata is a first step towards addressing unique nutritional needs and health challenges at different life stages.

This section discusses the role of additional aspects of nutrition, education, assessment and monitoring of potential benefits of nutrients and bioactive substances to optimize health outcomes and highlights challenges, opportunities, and future directions in life-stage guided approaches to nutrition and supplementation.

The Role of Food-Sourced Nutrients in Precision Nutrition

Nutrition from food, especially whole foods, remains the preferred way for most individuals to fulfill their nutritional requirements. Whole foods provide a range of nutrients, including essential vitamins, minerals, fiber, and bioactive compounds, which interact to support health. Complex interactions among these components in whole foods enhance nutrient absorption and utilization. However, in practice, many individuals find it challenging to meet their nutritional requirements through food alone. Multiple factors such as limited understanding of nutritional principles, poor access to high-quality foods, lifestyle and dietary choices or restrictions, or certain health conditions, can impede one’s ability to achieve optimal nutrient intake. In such cases, dietary supplements may serve as a valuable adjunct, helping individuals to fill specific nutritional gaps [38].

The principles of life-stage guided nutrition that we have discussed also apply to food-focused approaches, as is apparent in national nutritional policy in Korea and other countries. For example, health management guidance targeted to Korean individuals 50-64 years old places a strong emphasis on food and healthy eating habits for adequate nutritional intake to support healthy aging [37]. In older community-dwelling Korean adults, individualized educational and support programs have been found to positively influence dietary habits, nutritional knowledge and nutritional status [126]. Additionally, emerging approaches such as culinary medicine or culinary nutrition education for health professionals represent innovative strategies that could help them guide individuals toward better diets that meet their specific needs and perhaps reduce the burden of nutrition-related chronic diseases as people age [127,128]. Importantly, besides targeting conventional education areas such as nutritional knowledge, approaches like culinary-nutritional education also emphasize training in skills such as cooking, that could help individuals achieve and sustain healthier dietary patterns [128].

Better Assessment and Monitoring of Nutritional Needs

Dietary supplementation use should be guided by nutritional needs, considering factors such as nutrient status, sufficiency, and absorption/bioavailability. Establishing reference ranges for biomarkers of nutrient status remains a challenge for many micronutrients, with a lack of consensus on reliable, meaningful indicators [129]. Additionally, noninvasive and readily accessible measures to assess nutrient status are scarce, highlighting opportunities for research into developing more comprehensive and universally applicable standards for assessing nutrient status and deriving population or subgroup norms.

Appropriate biomarkers and surrogate endpoints allow the effects of supplementation on specific processes and health outcomes to be determined [130]. For example, serum 25-hydroxyvitamin D levels, or biomarkers like osteocalcin and C-telopeptide, can be used to assess the impact of vitamin D and calcium supplementation on bone health, but such biomarkers are not available for many important health outcomes. Monitoring of surrogate endpoints that are well correlated with outcomes, such as blood pressure, supports intermediate assessments of supplementation effects on health and disease risk. Additionally, advancements in diagnostic technologies may improve biomarker testing to identify specific nutrient deficiencies and track changes through time. This would facilitate the tailoring of supplementation based on individuals’ biomarker profiles. With the increasing availability of fitness trackers, mobile apps, and wearable devices, individuals can continuously monitor health-related indicators such as physical activity, sleep, and vital signs [131], and artificial intelligence-powered advancements may facilitate precise, real-time evaluation of these health data [3].

Practical Considerations: How “Precise” Should We Be?

Although fully personalized nutrition may not be desired or achievable for everyone, targeted precision approaches remain valuable in the context of variable resource levels and consumer choice. The level of precision required depends upon various factors, including the specific population group being targeted. For generally healthy individuals, a basic MVMS formulation providing 100% of RDAs or adequate intake levels may be sufficient to address potential nutrient gaps. However, certain population subgroups may have unique nutritional needs that warrant a more tailored approach. For instance, pregnant women and older adults may have specific and/or increased nutrient requirements and would benefit from formulations that cater to their specific needs. Highly personalized precision nutrition strategies, such as those incorporating genetic or metabolomic data, may be relevant for some individuals with specific health conditions or risks. Such targeted interventions address individual variations in nutrient metabolism and absorption, thereby optimizing the effectiveness and safety of supplementation.

Instead of overly precise formulations that may not be desired, necessary or cost-effective for the general population, balanced approaches that consider relevant factors (age, sex, life stage, etc.) are preferable. These would promote adequate nutrient intake while minimizing the risk of excessive consumption. By striking a balance between precision and practicality, tailored MVMS could effectively support overall health and well-being across a range of population groups. For example, developing supplement formulations for men and women by life stage can ensure better alignment of nutrient intake with specific needs. This targeted supplementation approach could support healthier dietary patterns and lifestyle habits, helping individuals achieve their health goals.

Navigating Challenges and Opportunities

Several challenges arise when considering life-stage nutrition and MVMS. Firstly, effectively communicating the benefits of dietary supplements to consumers requires clear and accurate messaging. The large volume of nutrition information available can overwhelm consumers, leading to confusion and decision paralysis. Overcoming this overwhelming information and misinformation, as well as promoting transparency about product efficacy, is essential for building consumer trust. A related concern is consumer fatigue. The proliferation of individually packed supplements and multivitamins on the market can overwhelm consumers. With numerous options available, consumers may experience decision fatigue and uncertainty about which supplements to choose. As with many health practices, such as exercise, adherence represents another challenge, as incorporating supplements into daily routines can be difficult for consumers. Factors such as taste preferences, dosing frequency, and lifestyle habits may influence adherence to supplementation regimens. Complex dosing regimens add to this challenge, as some supplements require multiple daily doses, or have special dosing or dietary requirements. Managing this complexity can be challenging, particularly for individuals with busy lifestyles or medical conditions.

Stratified, simplified once-daily formulations offer a potential solution to these challenges. Manufacturers can develop once-daily MVMS, reducing the complexity of dosing regimens and improving convenience and adherence for consumers. Life-stage nutrition principles can be leveraged to create once-daily MVMS with customized nutrient profiles to optimize health outcomes. Life-stage-specific MVMS also offers a way to address consumers’ health priorities and preferences. A 2021 Korean study on consumer characteristics related to health-functional food intake found that younger adults tended to consume multivitamins in order to support general health and immunity, whereas older adults were more likely to consume supplements like EPA/DHA-containing products and lutein, to maintain eye health and cardiovascular health and mitigate effects of age-related conditions [18]. Women were more likely than men to consume single and multi-vitamins, lutein, and EPA/DHA-containing products [18]. Market research findings from 2023 indicate that Korean consumers consider MVMS that are tailored ‘for each age group’ and ‘for every lifecycle’ to be highly relevant to their health goals [132]. By customizing life-stage specific MVMS, manufacturers can better address diverse health priorities within the population.

Lastly, improving consumer education and trust in MVMS is essential. Education initiatives can raise awareness about the role that life-stage nutrition and MVMS could play in supporting optimal health. Clear information can empower consumers to make informed decisions. Building partnerships with healthcare providers, such as physicians, dietitians, and pharmacists, can enhance consumer awareness of effective supplementation, and improve customization of supplementation formulations/regimens to build a solid foundation for health.

## Conclusions

Expanding on the concept of life-stage nutrition developed in this review, a more inclusive life-stage guided nutrition approach would ideally consider additional factors such as body composition, lifestyle and activity levels, dietary preferences, and social/cultural influences. Incorporating social determinants of health is important to ensure recommendations align not only with biological attributes but also with cultural, community, and economic contexts. Similarly, behavior significantly impacts health outcomes and is measurable, like biological attributes. Precision nutrition strategies can thus draw from biological evidence (differential responses to foods/nutrients based on genotypic or phenotypic characteristics), as well as analysis of behaviors, preferences, barriers, and objectives, to motivate individuals to make changes that will promote long-term health and well-being.

Our exploration of precision nutrition through a life-stage approach supports its value in promoting optimal health. With the starting point of age and gender, supported by appropriate biomarker assessment, dietary and supplementation strategies can be tailored to address unique nutritional needs and health challenges across the lifespan.

## References

[1] Bruins MJ, Van Dael P, Eggersdorfer M (2019). The role of nutrients in reducing the risk for noncommunicable diseases during aging. Nutrients.

[2] Hoffman DJ, Reynolds RM, Hardy DB (2017). Developmental origins of health and disease: current knowledge and potential mechanisms. Nutr Rev.

[3] Ordovas JM, Ferguson LR, Tai ES, Mathers JC (2018). Personalised nutrition and health. BMJ.

[4] Ferguson LR, De Caterina R, Görman U (2016). Guide and position of the International Society of Nutrigenetics/Nutrigenomics on Personalised Nutrition: Part 1 - fields of precision nutrition. J Nutrigenet Nutrigenomics.

[5] Livingstone KM, Ramos-Lopez O, Pérusse L, Kato H, Ordovas JM, Martínez JA (2022). Precision nutrition: a review of current approaches and future endeavors. Trends Food Sci Technol.

[6] Mompeo O, Freidin MB, Gibson R (2022). Genome-wide association analysis of over 170,000 individuals from the UK Biobank identifies seven loci associated with Dietary Approaches to Stop Hypertension (DASH) diet. Nutrients.

[7] de Toro-Martín J, Arsenault BJ, Després JP, Vohl MC (2017). Precision nutrition: a review of personalized nutritional approaches for the prevention and management of metabolic syndrome. Nutrients.

[8] Biesalski HK, Tinz J (2018). Micronutrients in the life cycle: requirements and sufficient supply. NFS Journal.

[9] Bundy DAP, Silva ND, Horton S, Jamison DT, Patton GC (2017). Chapter 1. Child and adolescent health and development: realizing neglected potential. Child and Adolescent Health and Development (3rd edition).

[10] Lassi Z, Moin A, Bhutta Z (2017). Chapter 11. Nutrition in middle childhood and adolescence. Child and Adolescent Health and Development (3rd edition).

[11] Arnett JJ, Zukauskiene R, Sugimura K (2014). The new life stage of emerging adulthood at ages 18-29 years: implications for mental health. Lancet Psychiatry.

[12] Lachman ME, Teshale S, Agrigoroaei S (2015). Midlife as a pivotal period in the life course: balancing growth and decline at the crossroads of youth and old age. Int J Behav Dev.

[13] (2024). Erik Erikson's stages of psychosocial development. https://www.simplypsychology.org/Erik-Erikson.html.

[14] Mehta CM, Arnett JJ, Palmer CG, Nelson LJ (2020). Established adulthood: a new conception of ages 30 to 45. Am Psychol.

[15] Figueira I, Fernandes A, Mladenovic Djordjevic A (2016). Interventions for age-related diseases: shifting the paradigm. Mech Ageing Dev.

[16] Diaz T, Strong KL, Cao B (2021). A call for standardised age-disaggregated health data. Lancet Healthy Longev.

[17] (2024). Age standardization of rates: a new WHO standard. https://cdn.who.int/media/docs/default-source/gho-documents/global-health-estimates/gpe_discussion_paper_series_paper31_2001_age_standardization_rates.pdf..

[18] Kim D, Ji I, Han K, Ng'ombe JN (2021). Effects of consumer characteristics on the intake of health functional foods: implications for National Health Expenditure Savings. Food Suppl Biomater Health.

[19] Kwon HY (2023). Who persistently consumes dietary supplements? A multifaceted analysis using South Korea's nationally representative health and nutrition examination survey data. Front Nutr.

[20] Lee JS, Kim J (2009). Factors affecting the use of dietary supplements by Korean adults: data from the Korean National Health and Nutrition Examination Survey III. J Am Diet Assoc.

[21] Hwang MY, Hong J (2023). Effect of dietary supplements on vitamin and mineral intake among Koreans: Data From the 2018-2020 Korea National Health and Nutrition Examination Survey. Food Suppl Biomater Health.

[22] Choi J, Yun EK, Byun HM (2023). Identifying patterns of lifestyle behaviours linked to sociodemographic characteristics and health conditions among young adults in South Korea. J Adv Nurs.

[23] Chung N, Park HY, Park MY (2017). Association of daily physical activity level with health-related factors by gender and age-specific differences among Korean adults based on the sixth (2014-2015) Korea national health and nutrition examination survey. J Exerc Nutrition Biochem.

[24] Hwang J-Y, Kim Y, Lee HS (2022). The development of resources for the application of 2020 Dietary Reference Intakes for Koreans. J Nutr Health.

[25] The Korean Nutrition Society (2020). 2020 Dietary Reference Intakes for Koreans. The Korean Ministry of Health and Welfare, Nutrition Society.

[26] Kwon KM, Lee JS, Jeon NE, Kim YH (2017). Factors associated with health-related quality of life in Koreans aged over 50 Years: the fourth and fifth Korea National Health and Nutrition Examination Survey. Health Qual Life Outcomes.

[27] Jang HY, Ahn JW, Jeon MK (2018). Factors affecting body image discordance amongst Korean adults aged 19-39 years. Osong Public Health Res Perspect.

[28] Lee MR, Jung SM, Bang H, Kim HS, Kim YB (2018). The association between muscular strength and depression in Korean adults: a cross-sectional analysis of the sixth Korea National Health and Nutrition Examination Survey (KNHANES VI) 2014. BMC Public Health.

[29] Lee SJ, Ryu HK (2022). Association between drinking behaviors and components of metabolic syndrome in subjects in their 20s and 30s: data obtained from the Korea National Health and Nutrition Examination Survey (2016-2018). Nutr Res Pract.

[30] Halloran EC (2024). Adult development and associated health risks. J Patient Cent Res Rev.

[31] Liu K, Daviglus ML, Loria CM, Colangelo LA, Spring B, Moller AC, Lloyd-Jones DM (2012). Healthy lifestyle through young adulthood and the presence of low cardiovascular disease risk profile in middle age: the Coronary Artery Risk Development in (Young) Adults (CARDIA) study. Circulation.

[32] Nagata JM, Vittinghoff E, Gabriel KP (2022). Physical activity from young adulthood to middle age and premature cardiovascular disease events: a 30-year population-based cohort study. Int J Behav Nutr Phys Act.

[33] Park K-R, Cho Y-C (2016). Prevalence rates of risk factors of metabolic syndrome, and its related with obesity indices among the health checkup examinees. Journal of the Korea Academia-Industrial Cooperation Society.

[34] Park HS, Oh SW, Cho SI, Choi WH, Kim YS (2004). The metabolic syndrome and associated lifestyle factors among South Korean adults. Int J Epidemiol.

[35] Chu JE, Lee JM, Cho H-I, Park YJ (2013). Relationships between obesity, blood and urinary compositions, and dietary habits and depressed mood in Koreans at the age of 40, a life transition period. J Nutr Health.

[36] Kim HS, Shin DW, Lee WC, Kim YT, Cho B (2012). National screening program for transitional ages in Korea: a new screening for strengthening primary prevention and follow-up care. J Korean Med Sci.

[37] Ministry of Food and Drug Safety Korea (2024). Customized Diet Management Guide for Middle-aged People (50-64 Years Old) (for the General Public). https://foodsafetykorea.go.kr/portal/board/boardDetail.do?menu_no=4852&bbs_no=NUTRI01&ntctxt_no=1083151&menu_grp=MENU_NEW03.

[38] Dietary Guidelines Advisory Committee: Scientific Report of the 2020 Dietary Guidelines Advisory Committee. Washington, DC DC (2024). Scientific Report of the 2020 Dietary Guidelines Advisory Committee, Washington DC, USA. https://www.dietaryguidelines.gov/sites/default/files/2020-07/ScientificReport_of_the_2020DietaryGuidelinesAdvisoryCommittee_first-print.pdf.

[39] Blumberg JB, Cena H, Barr SI (2018). The use of multivitamin/multimineral supplements: a modified Delphi consensus panel report. Clin Ther.

[40] Franceschi C, Garagnani P, Morsiani C (2018). The continuum of aging and age-related diseases: common mechanisms but different rates. Front Med (Lausanne).

[41] Niccoli T, Partridge L (2012). Ageing as a risk factor for disease. Curr Biol.

[42] Tuttle CS, Waaijer ME, Slee-Valentijn MS, Stijnen T, Westendorp R, Maier AB (2020). Cellular senescence and chronological age in various human tissues: a systematic review and meta-analysis. Aging Cell.

[43] Hägg S, Jylhävä J (2021). Sex differences in biological aging with a focus on human studies. Elife.

[44] Mauvais-Jarvis F, Bairey Merz N, Barnes PJ (2020). Sex and gender: modifiers of health, disease, and medicine. Lancet.

[45] Riggs BL, Khosla S, Melton LJ 3rd (2002). Sex steroids and the construction and conservation of the adult skeleton. Endocr Rev.

[46] You H, Shin HR, Song S, Ly SY (2022). Vitamin D intake and bone mineral density in Korean adults: analysis of the 2009-2011 Korea National Health and Nutrition Examination Survey. Nutr Res Pract.

[47] Shin HR, Lee YJ, Ly SY (2023). Optimal serum 25(OH)D levels and vitamin D intake in Korean postmenopausal women. Nutrients.

[48] Shin HR, Park HJ, Ly SY (2022). Optimal serum 25(OH)D level and vitamin D intake in young Korean women. Nutrients.

[49] Lee JK, Tran TM, Choi E (2024). Association between daily dietary calcium intake and the risk of cardiovascular disease (CVD) in postmenopausal Korean women. Nutrients.

[50] Lee YK, Chang JS, Min YK, Byun DW, Park Y, Ha YC (2017). Low calcium and vitamin D intake in Korean women over 50 years of age. J Bone Miner Metab.

[51] Yoo KO, Kim MJ, Ly SY (2019). Association between vitamin D intake and bone mineral density in Koreans aged ≥ 50 years: analysis of the 2009 Korea National Health and Nutrition Examination Survey using a newly established vitamin D database. Nutr Res Pract.

[52] Yelland S, Steenson S, Creedon A, Stanner S (2023). The role of diet in managing menopausal symptoms: a narrative review. Nutr Bull.

[53] Silva TR, Oppermann K, Reis FM, Spritzer PM (2021). Nutrition in menopausal women: a narrative review. Nutrients.

[54] Becker U, Deis A, Sørensen TI (1996). Prediction of risk of liver disease by alcohol intake, sex, and age: a prospective population study. Hepatology.

[55] Osna NA, Donohue TM Jr, Kharbanda KK (2017). Alcoholic liver disease: Pathogenesis and current management. Alcohol Res.

[56] Van Dael P (2021). Role of n-3 long-chain polyunsaturated fatty acids in human nutrition and health: review of recent studies and recommendations. Nutr Res Pract.

[57] Calder PC (2017). Omega-3 fatty acids and inflammatory processes: from molecules to man. Biochem Soc Trans.

[58] Bae JH, Lim H, Lim S (2023). The potential cardiometabolic effects of long-chain omega-3 polyunsaturated fatty acids: recent updates and controversies. Adv Nutr.

[59] Michaeloudes C, Christodoulides S, Christodoulou P, Kyriakou TC, Patrikios I, Stephanou A (2023). Variability in the clinical effects of the omega-3 polyunsaturated fatty acids DHA and EPA in cardiovascular disease-possible causes and future considerations. Nutrients.

[60] Moosavi D, Vuckovic I, Kunz HE, Lanza IR (2022). A randomized trial of omega-3 fatty acid supplementation and circulating lipoprotein subclasses in healthy older adults. J Nutr.

[61] Endo J, Arita M (2016). Cardioprotective mechanism of omega-3 polyunsaturated fatty acids. J Cardiol.

[62] Arabi SM, Bahari H, Chambari M, Bahrami LS, Mohaildeen Gubari MI, Watts GF, Sahebkar A (2024). Omega-3 fatty acids and endothelial function: a GRADE-assessed systematic review and meta-analysis. Eur J Clin Invest.

[63] Campbell F, Dickinson HO, Critchley JA, Ford GA, Bradburn M (2013). A systematic review of fish-oil supplements for the prevention and treatment of hypertension. Eur J Prev Cardiol.

[64] Elagizi A, Lavie CJ, O'Keefe E, Marshall K, O'Keefe JH, Milani RV (2021). An update on omega-3 polyunsaturated fatty acids and cardiovascular health. Nutrients.

[65] Bernasconi AA, Wiest MM, Lavie CJ, Milani RV, Laukkanen JA (2021). Effect of omega-3 dosage on cardiovascular outcomes: an updated meta-analysis and meta-regression of interventional trials. Mayo Clin Proc.

[66] (2024). Food and Agriculture Organization of United Nations. FAO food and nutrition paper: Fats and fatty acids in human nutrition.. Geneva.

[67] EFSA EFSA (2010). Scientific opinion on dietary reference values for fats, including saturated fatty acids, polyunsaturated fatty acids, monounsaturated fatty acids, trans fatty acids, and cholesterol. EFSA Journal.

[68] Park Y (2022). Dietary reference intake of n-3 polyunsaturated fatty acids for Koreans. Nutr Res Pract.

[69] Thompson M, Hein N, Hanson C (2019). Omega-3 fatty acid intake by age, gender, and pregnancy status in the United States: National Health and Nutrition Examination Survey 2003⁻2014. Nutrients.

[70] Mavar M, Sorić T, Bagarić E, Sarić A, Matek Sarić M (2024). The power of vitamin D: Is the future in precision nutrition through personalized supplementation plans?. Nutrients.

[71] Beto JA (2015). The role of calcium in human aging. Clin Nutr Res.

[72] Veldurthy V, Wei R, Oz L, Dhawan P, Jeon YH, Christakos S (2016). Vitamin D, calcium homeostasis and aging. Bone Res.

[73] Kenkre JS, Bassett J (2018). The bone remodelling cycle. Ann Clin Biochem.

[74] Institute of Medicine (US) Committee to Review Dietary Reference Intakes for Vitamin D and Calcium (2011). Dietary Reference Intakes for Calcium and Vitamin D.

[75] (2024). The Scientific Advisory Committee on Nutrition (SACN) recommendations on vitamin D. https://www.gov.uk/government/publications/sacn-vitamin-d-and-health-report.

[76] Holick MF, Binkley NC, Bischoff-Ferrari HA (2011). Evaluation, treatment, and prevention of vitamin D deficiency: an Endocrine Society clinical practice guideline. J Clin Endocrinol Metab.

[77] Salovaara K, Tuppurainen M, Kärkkäinen M (2010). Effect of vitamin D(3) and calcium on fracture risk in 65- to 71-year-old women: a population-based 3-year randomized, controlled trial--the OSTPRE-FPS. J Bone Miner Res.

[78] Choe SA, Sung J (2020). Trends of premature and early menopause: a comparative study of the US National Health and Nutrition Examination Survey and the Korea National Health and Nutrition Examination Survey. J Korean Med Sci.

[79] Liu Y, Le S, Liu Y (2022). The effect of calcium supplementation in people under 35 years old: a systematic review and meta-analysis of randomized controlled trials. Elife.

[80] Méndez-Sánchez L, Clark P, Winzenberg TM, Tugwell P, Correa-Burrows P, Costello R (2023). Calcium and vitamin D for increasing bone mineral density in premenopausal women. Cochrane Database Syst Rev.

[81] Weaver CM, Alexander DD, Boushey CJ (2016). Calcium plus vitamin D supplementation and risk of fractures: an updated meta-analysis from the National Osteoporosis Foundation. Osteoporos Int.

[82] Avenell A, Mak JC, O'Connell D (2014). Vitamin D and vitamin D analogues for preventing fractures in post-menopausal women and older men. Cochrane Database Syst Rev.

[83] Li S, Xi C, Li L (2021). Comparisons of different vitamin D supplementation for prevention of osteoporotic fractures: a Bayesian network meta-analysis and meta-regression of randomised controlled trials. Int J Food Sci Nutr.

[84] Manoj P, Derwin R, George S (2023). What is the impact of daily oral supplementation of vitamin D3 (cholecalciferol) plus calcium on the incidence of hip fracture in older people? A systematic review and meta-analysis. Int J Older People Nurs.

[85] Wong T, Bloomer RJ, Benjamin RL, Buddington RK (2017). Small intestinal absorption of methylsulfonylmethane (MSM) and accumulation of the sulfur moiety in selected tissues of mice. Nutrients.

[86] van de Poll MC, Dejong CH, Soeters PB (2006). Adequate range for sulfur-containing amino acids and biomarkers for their excess: lessons from enteral and parenteral nutrition. J Nutr.

[87] Nimni ME, Han B, Cordoba F (2007). Are we getting enough sulfur in our diet?. Nutr Metab (Lond).

[88] Butawan M, Benjamin RL, Bloomer RJ (2017). Methylsulfonylmethane: applications and safety of a novel dietary supplement. Nutrients.

[89] Brien S, Prescott P, Lewith G (2011). Meta-analysis of the related nutritional supplements dimethyl sulfoxide and methylsulfonylmethane in the treatment of osteoarthritis of the knee. Evid Based Complement Alternat Med.

[90] Kim LS, Axelrod LJ, Howard P, Buratovich N, Waters RF (2006). Efficacy of methylsulfonylmethane (MSM) in osteoarthritis pain of the knee: a pilot clinical trial. Osteoarthritis Cartilage.

[91] Debbi EM, Agar G, Fichman G (2011). Efficacy of methylsulfonylmethane supplementation on osteoarthritis of the knee: a randomized controlled study. BMC Complement Altern Med.

[92] Toguchi A, Noguchi N, Kanno T, Yamada A (2023). Methylsulfonylmethane improves knee quality of life in participants with mild knee pain: a randomized, double-blind, placebo-controlled trial. Nutrients.

[93] Swaminathan R (2003). Magnesium metabolism and its disorders. Clin Biochem Rev.

[94] Souza AC, Vasconcelos AR, Dias DD, Komoni G, Name JJ (2023). The integral role of magnesium in muscle integrity and aging: a comprehensive review. Nutrients.

[95] de Baaij JH, Hoenderop JG, Bindels RJ (2015). Magnesium in man: implications for health and disease. Physiol Rev.

[96] Institute of Medicine (US) Standing Committee on the Scientific Evaluation of Dietary Reference Intakes (1997). Dietary Reference Intakes for Calcium, Phosphorus, Magnesium, Vitamin D, and Fluoride.

[97] Wang R, Chen C, Liu W, Zhou T, Xun P, He K, Chen P (2017). The effect of magnesium supplementation on muscle fitness: a meta-analysis and systematic review. Magnes Res.

[98] Andretta A, Dias Batista E, Madalozzo Schieferdecker ME, Rasmussen Petterle R, Boguszewski CL, Dos Santos Paiva E (2019). Relation between magnesium and calcium and parameters of pain, quality of life and depression in women with fibromyalgia. Adv Rheumatol.

[99] Bagis S, Karabiber M, As I, Tamer L, Erdogan C, Atalay A (2013). Is magnesium citrate treatment effective on pain, clinical parameters and functional status in patients with fibromyalgia?. Rheumatol Int.

[100] Veronese N, Pizzol D, Smith L, Dominguez LJ, Barbagallo M (2022). Effect of magnesium supplementation on inflammatory parameters: a meta-analysis of randomized controlled trials. Nutrients.

[101] Loskutova E, Nolan J, Howard A, Beatty S (2013). Macular pigment and its contribution to vision. Nutrients.

[102] Whitehead AJ, Mares JA, Danis RP (2006). Macular pigment: a review of current knowledge. Arch Ophthalmol.

[103] Hammond BR Jr, Wooten BR, Curran-Celentano J (2001). Carotenoids in the retina and lens: possible acute and chronic effects on human visual performance. Arch Biochem Biophys.

[104] Bernstein PS, Zhao DY, Wintch SW, Ermakov IV, McClane RW, Gellermann W (2002). Resonance Raman measurement of macular carotenoids in normal subjects and in age-related macular degeneration patients. Ophthalmology.

[105] Hong IH, Jung WH, Lee JH, Chang IB (2020). Macular pigment optical density in the Korean population: a cross sectional study. J Korean Med Sci.

[106] Weigert G, Kaya S, Pemp B (2011). Effects of lutein supplementation on macular pigment optical density and visual acuity in patients with age-related macular degeneration. Invest Ophthalmol Vis Sci.

[107] Stringham JM, Johnson EJ, Hammond BR (2019). Lutein across the lifespan: from childhood cognitive performance to the aging eye and brain. Curr Dev Nutr.

[108] Seddon JM, Ajani UA, Sperduto RD (1994). Dietary carotenoids, vitamins A, C, and E, and advanced age-related macular degeneration. Eye Disease Case-Control Study Group. JAMA.

[109] (2013). Lutein + zeaxanthin and omega-3 fatty acids for age-related macular degeneration: the Age-Related Eye Disease Study 2 (AREDS2) randomized clinical trial. JAMA.

[110] Lupton JR, Atkinson SA, Chang N (2014). Exploring the benefits and challenges of establishing a DRI-like process for bioactives. Eur J Nutr.

[111] Bhargava R, Kumar P (2015). Oral omega-3 fatty acid treatment for dry eye in contact lens wearers. Cornea.

[112] Bhargava R, Kumar P, Phogat H, Kaur A, Kumar M (2015). Oral omega-3 fatty acids treatment in computer vision syndrome related dry eye. Cont Lens Anterior Eye.

[113] Giannaccare G, Pellegrini M, Sebastiani S (2019). Efficacy of omega-3 fatty acid supplementation for treatment of dry eye disease: a meta-analysis of randomized clinical trials. Cornea.

[114] Dighriri IM, Alsubaie AM, Hakami FM (2022). Effects of omega-3 polyunsaturated fatty acids on brain functions: a systematic review. Cureus.

[115] Yang L, Zhao F, Sun Y, Wang Z, Li Q, Wang H, Lu Y (2024). N-3 polyunsaturated fatty acids in elderly with mild cognitive impairment: a systemic review and meta-analysis. J Alzheimers Dis.

[116] Suh SW, Lim E, Burm SY, Lee H, Bae JB, Han JW, Kim KW (2024). The influence of n-3 polyunsaturated fatty acids on cognitive function in individuals without dementia: a systematic review and dose-response meta-analysis. BMC Med.

[117] Jew S, Antoine J-M, Bourlioux P (2015). Nutrient essentiality revisited. J Funct Foods.

[118] Townsend JR, Kirby TO, Marshall TM, Church DD, Jajtner AR, Esposito R (2023). Foundational nutrition: implications for human health. Nutrients.

[119] Gao YR, Wang RP, Zhang L, Fan Y, Luan J, Liu Z, Yuan C (2023). Oral administration of hyaluronic acid to improve skin conditions via a randomized double-blind clinical test. Skin Res Technol.

[120] Gillessen A, Schmidt HH (2020). Silymarin as supportive treatment in liver diseases: a narrative review. Adv Ther.

[121] Malik A, Malik M, Qureshi S (2024). Effects of silymarin use on liver enzymes and metabolic factors in metabolic dysfunction-associated steatotic liver disease: a systematic review and meta-analysis. Can Liver J.

[122] Berry SJ, Coffey DS, Walsh PC, Ewing LL (1984). The development of human benign prostatic hyperplasia with age. J Urol.

[123] Liu M, Yin H, Wang F, Tian Y (2021). The therapeutic potential of saw palmetto extract in urological disorders. Nat Prod Commun.

[124] Kwon Y (2019). Use of saw palmetto (Serenoa repens) extract for benign prostatic hyperplasia. Food Sci Biotechnol.

[125] Ahn JA, Park J, Kim CJ (2018). Effects of an individualised nutritional education and support programme on dietary habits, nutritional knowledge and nutritional status of older adults living alone. J Clin Nurs.

[126] Hanson MA, Cooper C, Aihie Sayer A, Eendebak RJ, Clough GF, Beard JR (2016). Developmental aspects of a life course approach to healthy ageing. J Physiol.

[127] Asher RC, Shrewsbury VA, Bucher T, Collins CE (2022). Culinary medicine and culinary nutrition education for individuals with the capacity to influence health related behaviour change: a scoping review. J Hum Nutr Diet.

[128] Domper J, Gayoso L, Goni L, de la O V, Etxeberria U, Ruiz-Canela M (2024). Culinary medicine and healthy ageing: a comprehensive review. Nutr Res Rev.

[129] Adams SH, Anthony JC, Carvajal R (2020). Perspective: guiding principles for the implementation of personalized nutrition approaches that benefit health and function. Adv Nutr.

[130] Zhu Z, Li YL, Song S (2023). Editorial: biomarkers: precision nutrition in chronic diseases. Front Nutr.

[131] Berciano S, Figueiredo J, Brisbois TD (2022). Precision nutrition: maintaining scientific integrity while realizing market potential. Front Nutr.

[132] IPSOS IPSOS (2023). Report for Korea Centrum Inclusivity Volumetric Study 49.

